# An efficient pipeline for ancient DNA mapping and recovery of endogenous ancient DNA from whole‐genome sequencing data

**DOI:** 10.1002/ece3.7056

**Published:** 2020-12-21

**Authors:** Wenhao Xu, Yu Lin, Keliang Zhao, Haimeng Li, Yinping Tian, Jacob Njaramba Ngatia, Yue Ma, Sunil Kumar Sahu, Huabing Guo, Xiaosen Guo, Yan Chun Xu, Huan Liu, Karsten Kristiansen, Tianming Lan, Xinying Zhou

**Affiliations:** ^1^ Institute of Vertebrate Paleontology and Paleoanthropology Chinese Academy of Sciences Beijing China; ^2^ College of Informatics Huazhong Agricultural University Wuhan China; ^3^ State Key Laboratory of Agricultural Genomics BGI‐Shenzhen Shenzhen China; ^4^ Guangdong Provincial Key Laboratory of Genome Read and Write BGI‐Shenzhen Shenzhen China; ^5^ CAS Center for Excellence in Life and Paleoenvironment Beijing China; ^6^ School of Future Technology University of Chinese Academy of Sciences Beijing China; ^7^ College of Wildlife Resources Northeast Forestry University Harbin China; ^8^ Forest Inventory and Planning Institute of Jilin Province Changchun China; ^9^ Guangdong Provincial Academician Workstation of BGI Synthetic Genomics BGI‐Shenzhen Shenzhen China; ^10^ Department of Biology Laboratory of Genomics and Molecular Biomedicine University of Copenhagen Copenhagen Denmark

**Keywords:** ancient DNA, *BWA mem*, deamination, DNA damage, genome mapping

## Abstract

Ancient DNA research has developed rapidly over the past few decades due to improvements in PCR and next‐generation sequencing (NGS) technologies, but challenges still exist. One major challenge in relation to ancient DNA research is to recover genuine endogenous ancient DNA sequences from raw sequencing data. This is often difficult due to degradation of ancient DNA and high levels of contamination, especially homologous contamination that has extremely similar genetic background with that of the real ancient DNA. In this study, we collected whole‐genome sequencing (WGS) data from 6 ancient samples to compare different mapping algorithms. To further explore more effective methods to separate endogenous DNA from homologous contaminations, we attempted to recover reads based on ancient DNA specific characteristics of deamination, depurination, and DNA fragmentation with different parameters. We propose a quick and improved pipeline for separating endogenous ancient DNA while simultaneously decreasing homologous contaminations to very low proportions. Our goal in this research was to develop useful recommendations for ancient DNA mapping and for separation of endogenous DNA to facilitate future studies of ancient DNA.

## Graphical abstract

Our method for ancient DNA mapping and filtering contamination can decrease the contamination to very low level. We believe that the work we present can facilitate future studies of ancient DNA.

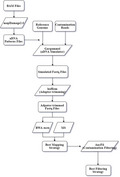

## 1 INTRODUCTION

Ancient DNA research provides direct evidence to reconstruct prehistoric biogeographies and biodiversities, which can further help to explain long‐standing questions in evolution, phylogeny, taxonomy, and adaptations (Chang et al., 2017; Delsuc et al., 2019; Palkopoulou et al., 2018; Sikora et al., 2019; Stoneking & Krause, 2011). Ancient DNA research has developed rapidly over the past thirty years due to improvements in PCR and next‐generation sequencing (NGS) technologies. The first successful attempt to extract ancient DNA was made by Higuchi et al. (1984), where DNA of *Equus quagga* was extracted from muscle and DNA fragments of 228 bp were amplified (Higuchi et al., 1984; Kefi, 2011). With advancements in biomolecular techniques, it is now possible to extract and amplify ancient DNA fragments from different ancient species and biological samples, including bones, teeth, soft tissue, fur, and subfossilized excrements (Kefi, 2011; Rizzi et al., 2012). Studies on ancient DNA were previously restricted to mitochondrial DNA and extremely short nuclear DNA fragments (Dabney, Knapp, et al., 2013; Kefi, 2011). However, the advent of NGS technology has enabled ancient DNA studies at the whole‐genome level. Consequently, the number of ancient DNA studies has increased exponentially in the last decade (Hofreiter et al., 2015). The first whole genome of woolly mammoth was sequenced in 2008 (Miller et al., 2008). Three Neanderthal genomes were also sequenced in 2010, revealing extensive gene flow to modern humans (Green et al., 2010). In 2012, the first high coverage genome (~30×) of Denisovans was published (Meyer et al., 2012). In 2015, Allentoft et al. (2015 sequenced 101 ancient humans at the whole‐genome level (Allentoft et al., 2015). At present, more than 1,100 ancient human and hominine genomes (Marciniak & Perry, 2017) and more than 300 ancient animal genomes (Fages et al., 2019; MacHugh et al., 2017; Palkopoulou et al., 2018) have been sequenced and published.

Although great breakthroughs have been made in ancient DNA extraction, library preparation and bioinformatics, some challenges remain (Gansauge & Meyer, 2013; Rohland et al., 2018; Schubert et al., 2012; Skoglund et al., 2014). Effective mapping and distinguishing of the present‐day DNA contaminations from endogenous ancient DNA are still complicated and difficult to perform, and need to be improved for ancient DNA analysis. It is particularly difficult to filter the present‐day human DNA contamination from ancient human or hominine DNA (Green et al., 2009; Richards et al., 1995). Ancient DNA is often degraded into very small fragments due to physical, chemical, or biological factors during a long‐term preservation in unfavorable conditions. These effects always leave valuable marks on ancient DNA to help us distinguish it from modern DNA, including C‐to‐T changes at the ends of ancient DNA fragments induced by deamination, high proportion of purine bases at the first physical position preceding ancient DNA fragments, and the severely fragmented nature (Skoglund et al., 2014; Stoneking & Krause, 2011).

Bioinformatics methods have been developed for mapping and separating endogenous ancient DNA from total ancient DNA (Schubert et al., 2012; Skoglund et al., 2014). In the mapping procedure for ancient DNA, the software BWA (Li & Durbin, 2009) with parameters set “*aln ‐l 1,024 ‐n 0.03*” is usually applied to map ancient sequencing data against the reference genome (Schubert et al., 2012). However, this process is time‐consuming. The newly developed method BWA *mem* with the *seed‐reseed‐extend* algorithm, provides improved efficiencies for mapping of ancient DNA (Li, 2013). Skoglund et al. (2014) developed PMDtools to separate genuine endogenous DNA from homologous contaminations. This method is effective in filtering modern human contaminated DNA from ancient human DNA. However, it is difficult for the PMDtools to set an appropriate threshold value of PMDS when contamination rates cannot be accurately evaluated. Besides, the power of PMDtools is further weakened for extremely young or old ancient samples.

In this study, we collected whole‐genome sequencing data generated by the Illumina Hiseq platform from 6 samples (representing three species) to optimize ancient DNA mapping. This step is critical to improving the mapping rate of endogenous ancient DNA. Since optimization of ancient DNA mapping may not only require filtering of present‐day contaminations from endogenous ancient DNA, we used our simulated data to further explored a more universal and effective filtration pipeline to filter present‐day contaminations based on ancient DNA cytosine deamination, depurination and fragmentation. The final recommendations presented here enabled reduction of modern human DNA contamination to an extremely low level while maintaining a high rate of endogenous DNA. We sought to develop mapping guidelines that, when coupled with screening recommendations to control for modern DNA contamination, could increase the effectiveness of future studies of ancient DNA.

## 2 MATERIALS AND METHODS

**FIGURE 1:**
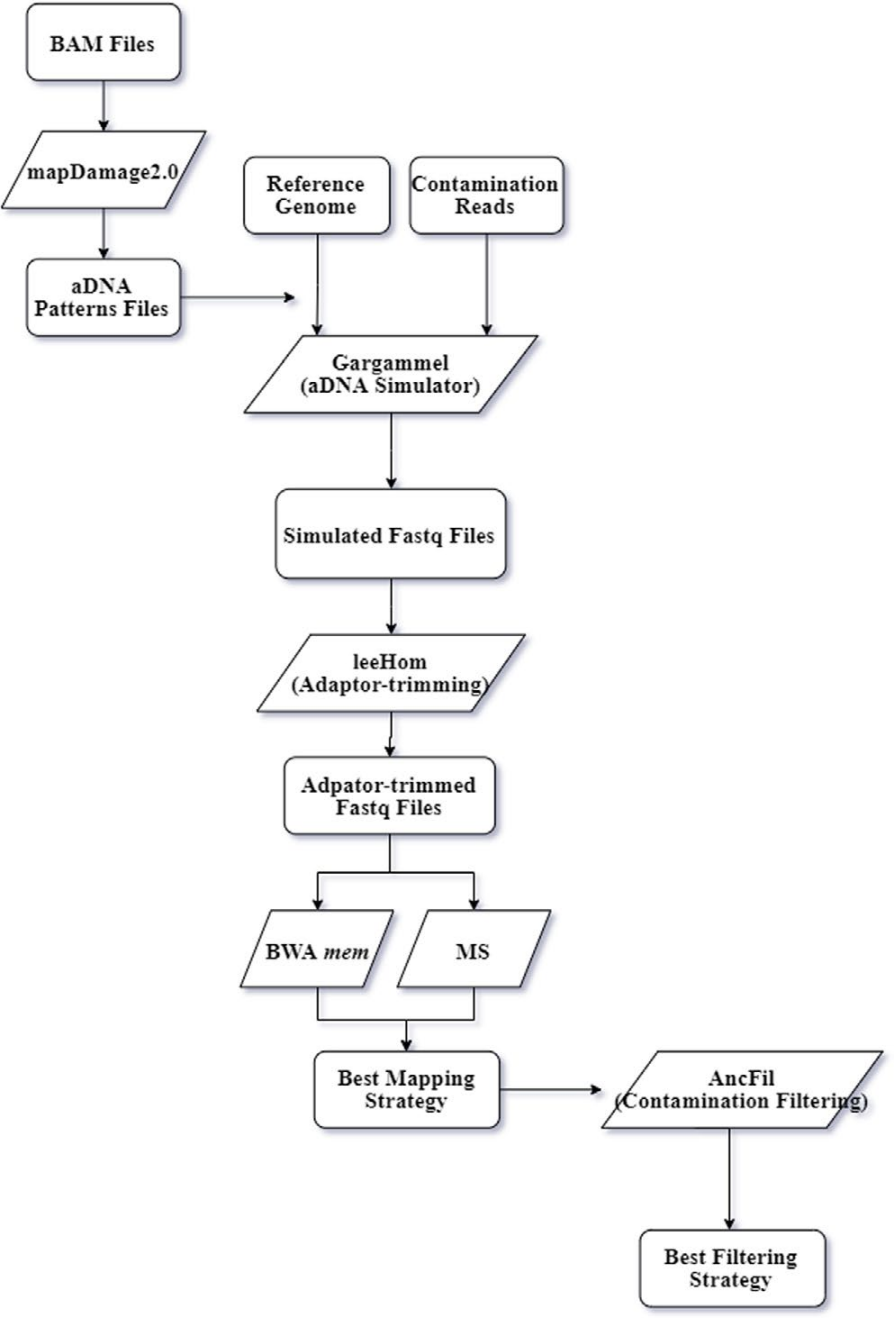
The experimental approach flow chart. Parallelogram box means the software we used

We used simulated ancient DNA data to find more effective strategies for mapping and separating endogenous ancient DNA from homologous contaminations. To make the design clearer, we drafted a flowchart to show our overall study design (Figure 1).

### 2.1 Samples and data resource

We investigated previously sequenced whole‐genome sequences from ancient animals. In total, we retrieved whole‐genome sequencing (WGS) data from 6 ancient samples derived from different age groups of three species, namely four ancient humans (*Homo sapiens*) (Fu et al., 2016; Sawyer et al., 2015; Schuenemann et al., 2017), one ancient goat (Daly et al., 2018), and one ancient aurochs (Park et al., 2015). The BAM files were downloaded from NCBI (https://www.ncbi.nlm.nih.gov/). The 6 samples were used to explore the methods for mapping and separating endogenous DNA (Table 1). The reference genomes for each species used for genome mapping are listed in Table S1.

**TABLE 1:** The description of samples and sequencing data used for simulating ancient DNA sequences

Species	Sample ID	Age (kyr BP)	Data sources	Reads number	Bases number	Average length of DNA fragments (bp)
*Homo sapiens*	JK2911	2.7	Schuenemann et al. (2017)	2.35E + 06	1.37E + 08	58.2
*Homo sapiens*	Villabruna	14	Fu et al. (2016)	1.22E + 07	6.70E + 08	54.9
*Homo sapiens*	AfontovaCava 3	17	Fu et al. (2016)	8.88E + 05	5.15E + 07	58.03
*Homo sapiens*	Denisova_8	>50	Sawyer et al. (2015)	8.26E + 05	3.69E + 07	44.6
*Bos primigenius*	British aurochs	6.7	Park et al., (2015)	7.51E + 07	3.48E + 09	46.29
*Capra aegagrus hircus*	Direkli5	11.5	Daly et al. (2018)	3.04E + 07	1.40E + 09	45.94

Abbreviation: BP, before present.

### 2.2 DNA damage analysis and ancient DNA simulation

Removing all contaminations present in real ancient data is often difficult and can lead to inaccurate evaluation. Therefore, we did not use real ancient sequencing data for analysis, but rather, we used simulated ancient sequences with the same damage parameters as those of the real data. With simulated data, it is possible to tag contamination and endogenous reads, which allows more reliable quantification of the effects. The most important thing for this ancient DNA simulation is to know the real state of the ancient DNA data we collected, especially investigating the real ancient DNA length distribution and the real proportion of deamination induced misincorporation (C‐to‐T and G‐to‐A) at ends of ancient DNA fragments. So we used mapDamage2.0 (Jonsson et al., 2013) to calculate the frequency of C‐to‐T and G‐to‐A changes at the ends of DNA fragments (misincorporation.txt) and length distribution (length_distribution.txt). To simulate real contaminations, we sequenced ancient DNA isolated from an ancient giant panda sample with ~100 years old (CNP0000732) by DIPSEQ‐T1 platform, and then filtered adaptors using Trimmomatic software based on adaptor sequences (>Adapter/1:AAGTCGGAGGCCAAGCGGTCTTAGGAAGACAA;>Adapter/2:AAGTCGGATCGTAGCCATGTCGTTCTGTGAGCCAAGGAGTTG). Raw reads were then mapped by BLASTing raw reads to the nucleotide database (Lan et al., 2019) to obtain all contaminated reads. This contamination consisted of DNA from more than twenty thousand modern species, mostly consisting of bacteria, and the top 10 contaminant species have been listed in the Figure S1. We also mapped raw sequencing reads to the giant panda reference genome to identify endogenous DNA. However, the rate of endogenous DNA is extremely low (<0.001%) and therefore not included in further ancient DNA simulation of the giant panda. Real contamination data were then added into simulated endogenous ancient DNA to test ancient genome mapping methods. We also added modern human DNA fragments (hg38 reference genome) into simulated ancient human DNA to explore the method for filtering homologue contamination. Finally, we used gargammel (Renaud et al., 2017) (perl gargammel.pl ‐n 1,000,000 ‐‐comp 0,cont_rate,endo_rate ‐f length_distribution.txt ‐mapdamage misincorporation_distribution.txt single_strand/double_strand ‐o data/simulation data/) to simulate FASTQ files including one million reads of ancient DNA sequences for our six ancient samples. Parameters in gargammel were strictly set based on results calculated by mapDamage2.0, in order to simulate the real state of these six samples. Nine different contamination rates (cont_rate) were simulated (20%, 40%, 60%, 80%, 90%, 95%, 99%, 99.5%, and 99.9%) (Table S3). And the gargammel works as following: Step 1: Reference genome sequences were cut into different length fragments, which are in consistent with real ancient DNA data length distribution (provided by mapDamage2.0). Step 2: The reference reads are added with different DNA damage characteristics which are in consistent with DNA damage patterns of real ancient DNA data (also provided by mapDamage2.0). And, contamination reads will not be cut into different length fragments and added with DNA damage patterns as step 1 and step 2. Contamination reads can be also generated from the reference genome of some target species. Step 3: Gargammel will generate a Fastq file including simulated real ancient data and simulated contamination data. And the percentage of simulated read ancient data in the Fastq file is consistent with the parameter “‐‐com” of gargammel (Figure 1).

### 2.3 Genome mapping of simulated ancient DNA

Ancient DNA damage, especially C‐to‐T changes, can result in mis‐mapping when ancient DNA fragments are mapped to reference genomes. Mapping methods and parameters used for modern DNA are not always suitable for ancient DNA (Schubert et al., 2012). We compared BWA *aln* and BWA *mem* to develop a more effective mapping strategy based on the characteristics of ancient DNA damage.

We used leeHom (Renaud et al., 2014) to trim adaptors and merge Illumina sequencing reads, and compared BWA *aln* (Version: 0.7.17) and BWA *mem* (Version: 0.7.17) to enhance mapping methods for ancient DNA. Here, “bwa *aln ‐l 1,024 ‐n* 0.03” (MS parameters) (Schubert et al., 2012) was compared with BWA *mem*. The valid mapping hits were defined as reads with endogenous ancient DNA tags (all simulated endogenous ancient DNA were tagged before mapping) and with a mapping quality higher than 30. Because it might be suitable for study of ancient DNA, the most important part of the BWA mem algorithm is the seed‐reseed‐extend strategy. When seeding, BWA will do exactly mapping by using part of the read length (19 bp in length when using the default parameter) on the reference genome based on FM‐index algorithm. A DNA fragment in the read will be chosen as a seed when its length and number of successful matches meet thresholds the user set. Then, the seed will be used to extend both in reads and reference genome to find global match based on Smith‐Waterman algorithm. This is mainly supported by two parameters including minimum seed length (parameter ‐*k*) and maximum seed length without reseeding (parameter ‐*r*). The parameter “‐*k*” controls the seeding function; seeding can accelerate genome mapping. Additionally, the algorithm searches for internal seeds inside a seed longer than x bp (x=[‐*k*] * [‐*r*]). We tried to optimize these two parameters to further explore more efficient mapping parameters for ancient DNA mapping. We tested BWA *mem* with ‐*k* (9/14/19/24/29) and ‐*r* (0.5/1/1.5/2/2.5) parameters. To evaluate mapping effectiveness, we defined three main criteria: (1) CRT: the contamination rate after treatment (the number of mapped contamination reads/ the number of mapped reads); (2) LRE: the loss rate of endogenous DNA (the number of unmapped endogenous ancient reads/the number of endogenous ancient reads); (3) MT: the running time of mapping.

### 2.4 Separating endogenous DNA from the contaminations

The unique ancient DNA characteristics, especially C‐to‐T and/or G‐to‐A changes at ends of DNA fragments help to improve filtering of contaminated present‐day DNA. We wrote a program named AncFil using Python (home page: https://github.com/tianminglan/AncFil) to explore a more universal and effective pipeline for separating endogenous ancient DNA from homologous contaminations. We first screened reads with at least “DeamNum” C‐to‐T or G‐to‐A mutations within the first or last “DetectRange” base pair at 3’ and/or 5’ ends (“DoubleOrSingle”). For “DeamNum” (the number of C‐to‐T or G‐to‐A mutations), we tested one, two and three. For “DetectRange” (the base number), we tested five, ten and fifteen, while for “DoubleOrSingle,” either 3’ or 5’ end (parameter “or”) and both ends (parameter “and”) were included. We explored all 18 possible screening conditions by adjusting the parameter combinations (“DeamNum,” “DetectRange,” “DoubleOrSingle”) (Table S2). One can test more possible conditions by adjusting parameters “‐DeamNum,” “‐DetectRange,” and “‐DoubleOrSingle.” Given that there is a natural tendency toward depurination at the 5’ ends of ancient DNA fragments ( Briggs et al., 2007), we screened reads with an A or G at the position preceding the first base of the 5’ end. Finally, we evaluated the effect of fragment length of ancient DNA on the separation of endogenous DNA. Here, two criteria were used to evaluate this pipeline: (1) CRT: the contamination rate after treatment (the number of contamination reads after filtering/the number of reads after filtering); (2) LRE: the loss rate of true endogenous DNA (the number of filtered endogenous ancient reads/the number of endogenous ancient reads before filtering);

Finally, PMDtools (Skoglund et al., 2014) were used to filter homologous contaminations using the same data and evaluating criteria used to evaluate our recommended method above. Meanwhile, “‐threshold” is one of the most important parameters in PMDtools for adjusting the strictness of the filtration. To make a complete comparison, we tested five threshold values (one, two, three, four, and five) to adjust the PMD scores by setting “‐threshold.”

## 3 RESULTS

### 3.1 Description of samples and the simulated data

We simulated a total of 90 ancient DNA datasets (Table S3) containing the same length distribution and damage patterns as the real dataset (Table 1). One million reads were finally simulated under each condition. The average length of ancient DNA data that was collected ranged from 45 bp to 58 bp (Table 1). The length distributions for most ancient DNA datasets ranged from 30 bp to 70 bp (Figure S2). The sample ages ranged from ~ 2.7 kyr BP (Before Present) to ~50 kyr BP, which provided a good basis to evaluate the influence of age on ancient DNA mapping and separation of homologous contaminations. The DNA damage analysis showed an obvious increase of deaminated substitutions with the frequency of C‐to‐T and G‐to‐A at the ends of DNA fragments ranging from 2% to 80% (Figure S3). Samples collected in our study included ancient DNA of different conditions, which enabled us to draw conclusions suitable for most ancient DNA samples.

### 3.2 Comparing different mapping algorithms on ancient DNA

(a) CRT: the contamination rate after treatment (the number of mapped contamination reads/ the number of mapped reads); (b) LRE: the loss rate of endogenous DNA (the number of unmapped endogenous ancient reads/ the number of endogenous ancient reads); (c) MT: the running time of mapping.

**FIGURE 2:**
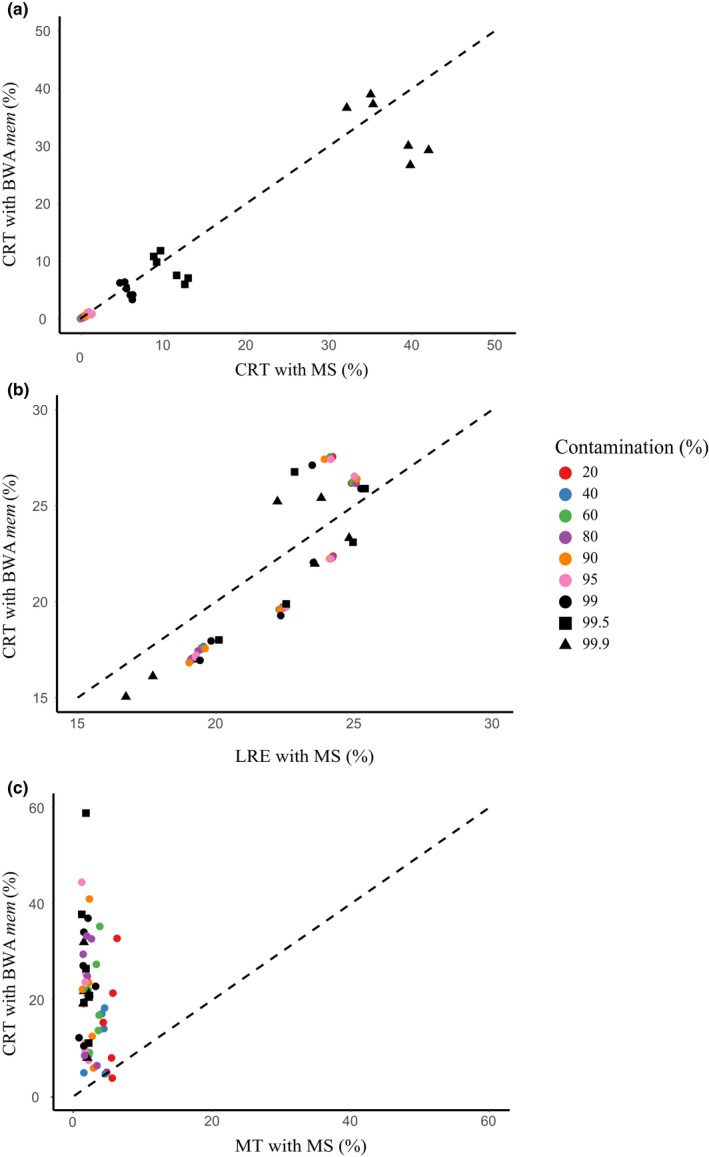
The differences between BWA *aln* with MS parameters and BWA *mem* with default parameters. (a) Comparison of CRT. (b) Comparison of LRE. (c) Comparison of MT

The comparison was achieved by calculating the contamination rate after treatment (CRT), the loss rate of endogenous DNA (LRE), and the running time of mapping (MT) for each dataset, and performing Repeated Measurement Analysis of Variance. The average and median values of CRT, LRE, and MT of the two algorithms with different contamination rates are shown in Table S4. The analysis showed no significant differences in CRT (*F = *1.42, *p* = .2870) and LRE (*F* = 0.44, *p* = .5344). However, significant differences were found in MT (*F* = 41.57, *p* = .0013) (Table S5) and BWA *aln* with the MS parameter requiring a multiple of 7.13 more times than BWA *mem* by default. We further evaluated the influence of different samples and different contamination rates on ancient DNA mapping. As shown in Table S6 and Figure 2, CRT levels were unchanged across different samples. The mean values of LRE were stable between contamination rates but not when the contamination rate was close to 100%.

**FIGURE 3:**
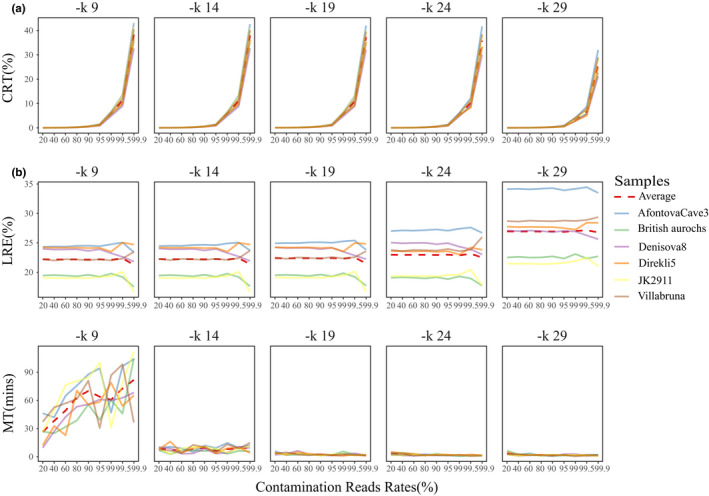
Comparison of BWA mem results with “‐*k*” parameters. (a) Comparison of CRT. (b) Comparison of LRE. (c) Comparison of MT

We calculated CRT, LRE, and MT using different parameters ‐*k* (9/14/19/24/29) (Figure 3) and performed Repeated Measurement Analysis of Variance Analysis to compare the results generated under different parameters. There were significant differences in CRT (*F* = 644.61, *p* < .0001), LRE (*F* = 17.99, *p* = .0057), and MT (*F* = 146.75, *p* < .0001) (Table S7). LRE was highest at ‐*k* = 29, and it increased as the value of k increased. LRE value decreased by ~0.20% from ‐*k* = 19 to ‐*k* = 9; however, this decrease continued to ~4.66% from ‐*k* = 29 to ‐*k* = 19, which was 22.3 times larger than that between ‐*k* = 19 and ‐*k* = 9 (Table S8). In addition, the running time significantly decreased from ‐*k* = 9 to ‐*k* = 19, but was relatively stable and slightly longer when ‐*k* was larger than 19 (Table S8, Figure 3).

**FIGURE 4:**
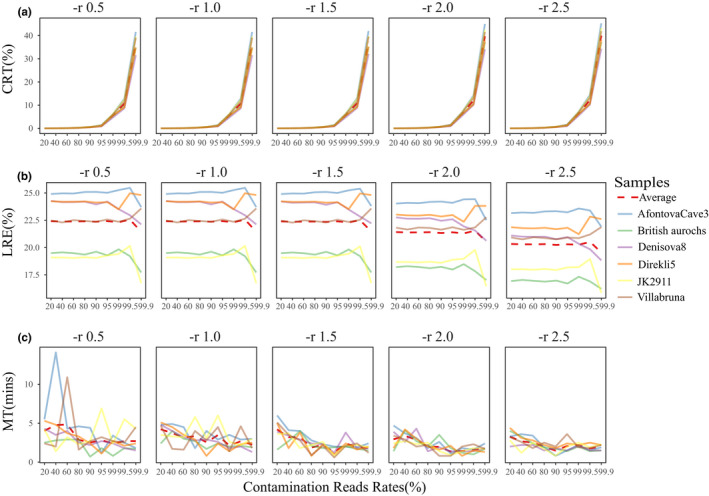
Comparison of “BWA mem” results with “‐*r*” parameters. (a) Comparison of CRT. (b) Comparison of LRE. (c) Comparison of MT

We evaluated the parameter ‐*r* (0.5/1/1.5/2/2.5) with the same method used to evaluate the parameter ‐*k* (Figure 4). Significant differences were found in CRT (*F* = 392.45, *p* < .0001), LRE (*F* = 45.11, *p* = .0010), and MT (*F* = 9.19, *p* = .0002) (Table S9). A significant decrease in LRE was recorded when the set values of “‐*r*” were greater than 1.5 and LRE reached the lowest level at ‐*r* = 2.5 (Table S10). Furthermore, the running time significantly decreased from ‐*r* = 0.5 to ‐*r* = 1.5, but it was relatively stable and slightly longer when ‐*r* was greater than 1.5 (Table S10, Figure 4).

### 3.3 Separation of endogenous DNA

**FIGURE 5:**
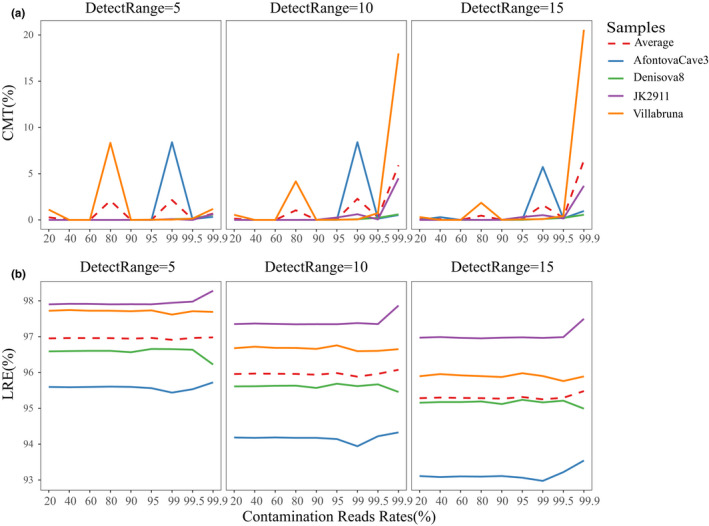
Comparison of deamination filtering with “‐DetectRange” parameters. (a) Comparison of CRT after filtering. (b) Comparison of LRE after filtering

Using unique ancient DNA characteristics, the homologous contamination rate was reduced to a very low level (Figure 5). The mean values of CRT and LRE are also shown in Table S11. No significant differences were found in CRT (*F* = 3.27, *p* = .1097) and LRE (*F* = 1.11, *p* = .3893) (Table S12).

When testing the influence of parameter “DeamNum” on separation of endogenous DNA, significant differences were found in CRT (*F* = 26.01, *p* = .0011) and LRE (*F* = 24.03, *p* = .0152) (Table S13). An increase in “DeamNum” resulted in lower values for CRT, but higher values for LRE (Figure S4). We also calculated CRT and LRE to evaluate the influence of the parameter “DoubleOrSingle” on ancient DNA mapping (Figure [Link], [Link]). Significant difference was found in values of CRT (*F* = 44.97, *p* = .0068) but not in LRE (*F* = 7.20, *p* = .0748) (Table S14). The result showed a decline of 90.27% in CRT when screening the reads with C‐to‐T or G‐to‐A on single end (‐DoubleOrSingle = or) compared to screening on both 3’ and 5’ ends (‐DoubleOrSingle = and) (Table S11). The homologous contamination rate was held to an average of 0.92% by using the filtering strategy with “‐DetectRange = 15 ‐DeamNum = 1 ‐DoubleOrSingle = or.”

We compared our method with PMDtools software. These two methods were run in parallel using the same dataset. The results generated by PMDtools with different parameters are shown in Table S15. To make the comparison fairer, LRE values of the tested dataset were kept similar in both our pipeline and PMDtools. CRT values did not differ (*Z* = −1.171*, p* = .241) between the two methods. However, the running time of our method was 15.43% of the runtime for PMDtools, and the difference was significant (Difference = 2.3mins, Z = −6.50, *p* = 8.28E^−11^). Although this comparison does not show that our tool outperforms PMDtools, it does demonstrate a fast and reliable complement to PMDtools.

We tried to screen reads with G or A residues preceding the first base at 5’ end. The average homologous contamination rate was 2.25% after filtering using depurination characteristic.

## 4 DISCUSSION

### 4.1 Comparing BWA *aln* and BWA *mem* to improve ancient DNA mapping

BWA *aln* uses backward search for exact matching and its seeding function allows differences in the first few tens of base pairs on a read to search inexact matching (Li & Durbin, 2009). This can accelerate genome mapping but it also increases the probability of incorrect alignments. Therefore, disabling the seed function often tends to be more effective in ancient DNA mapping (Schubert et al., 2012). BWA *mem*, however, uses a re‐seeding strategy to increase correct alignments when no maximal exact matches (SMEMs) can be found (Li, 2013). This could compensate the shortcoming of BWA *aln* mentioned above. In our experiment, no significant difference was found in CRT (*F = *1.42, *p* = .2870) between Schubert's method (Schubert et al., 2012) and the BWA *mem* algorithm. Consequently, the performance on ancient DNA mapping of BWA *mem* with default parameters (BWA *mem* ‐*k* 19 ‐*r* 1.5) was comparable to BWA *aln* with the MS parameters.

Additionally, the seed‐reseed‐extend strategy in BWA *mem* can help to accelerate the mapping process (Li & Durbin, 2009), and it resulted in a 87.70% decrease of MT compared to the BWA *aln* algorithm. Therefore, BWA *mem* can improve the accuracy of ancient genome mapping in a shorter time than that required for analysis using BWA *aln*.

Soft clipping (Langmead & Salzberg, 2012) means that some nucleotides at either terminal of the reads can be omitted as determined by the mapping scoring scheme. And it's one of most important issues to consider when using BWA *mem*. In our study, 7.9% of mapped reads were soft clipped during mapping and 6% of soft‐clipped reads contained C‐to‐T and/or G‐to‐A changes within soft‐clipped regions. In other words, only ~0.47% (7.9%*0.6%) mapped reads with damaged patterns were soft clipped, which was a small proportion when considering the large number of damaged endogenous DNA. With regard to hard clipping (Langmead & Salzberg, 2012), this means that some nucleotides at either terminal of reads can be omitted as determined by the mapping scoring scheme but the omitted nucleotides do not exist in the fragment. This is a special kind of soft clipping to mark the multiple mapping of a read. But only 0.0036% of mapped reads showed damaged patterns. Therefore, soft clipping only slightly impacted the filtering of endogenous DNA by using deamination characteristics. In summary, BWA *mem* performed as well as BWA *aln* with MS parameters in this study, but BWA *mem* required less running time (87.70% time) than did the BWA *aln* method. Taking all results into account, BWA *mem* performed better than BWA *aln*.‬

### 4.2 Exploring more accurate and effective mapping parameters of BWA *mem*

The parameters ‐*k* and ‐*r* are extremely important for the “seeding and reseeding” mapping stages in BWA *mem* (Li, 2013). The different parameter values of ‐*k* and ‐*r* could significantly affect CRT, LRE, and MT, indicating that we can obtain ancient DNA mapping results with a lower contamination rate by optimizing these parameter values (Tables S7, S9).

The BWA *mem* algorithm only found the maximal exact matches (SMEMs) in a read while seeding and this algorithm can trigger re‐seeding with SMEMs to reduce the loss of mis‐mapping if SMEMs are larger than [‐k*‐*r*] ( Li, 2013). The large [‐k*‐*r*] values meant fewer re‐seedings, which could accelerate the mapping process. This was consistent with the observation of runtime results. However, too long seeds could also make seed mapping against genomes more difficult and eventually more time‐consuming.

We also found that running time was more sensitive to changes in ‐*k* parameter than in ‐*r* (Table S8, Table S10, Figure 3, Figure 4), indicating that running time was mainly influenced by minimum seed length. The ‐*r* cannot affect seeding for SMEMs, but ‐*k* can influence both seeding and reseeding procedures (Li, 2013), which might be reason for their differing influence on running time. Finally, it took minimum runtime to save endogenous ancient DNA reads as much as possible when using “BWA mem ‐*k* = 19 ‐*r* = 2.5” for mapping of ancient DNA.

### 4.3 Improving the separation of endogenous DNA

Among all kinds of homologous contaminations, the present‐day human DNA is a very common contamination in ancient human DNA. This is because contaminations can easily be induced from the time samples are collected to the time DNA library preparation is performed. These homologous contaminations are extremely difficult to remove ( Skoglund et al., 2014).

**FIGURE 6:**
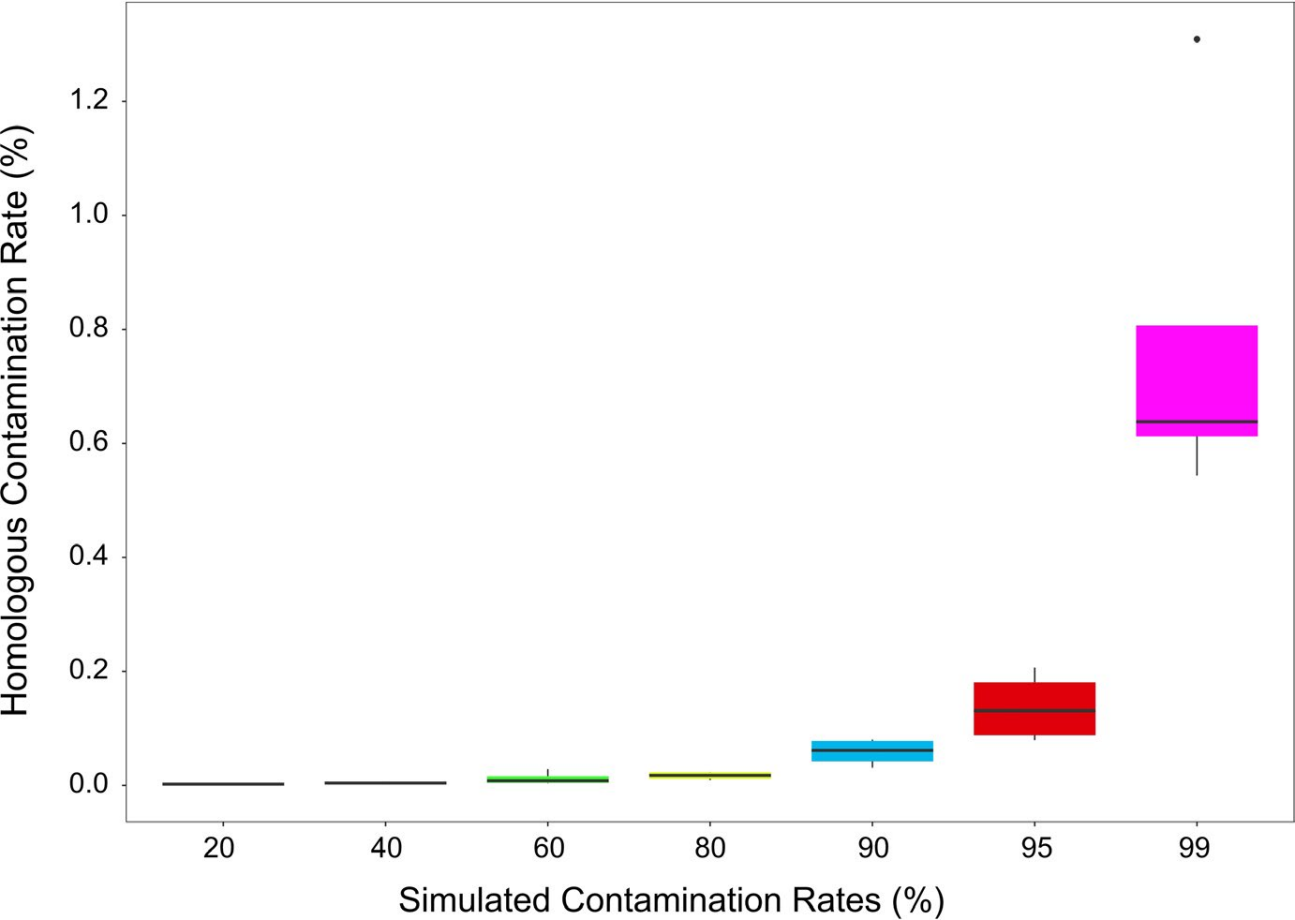
The homologous contamination rate after filtering by use of the AncFil with parameters –DeamNum = 15 –DetectRange = 1 –DoubleOrSingle = or. X‐axis means the rate of homologous contamination which was added in simulation data. Y‐axis means the rate of homologous contamination which was remaining after filtering

In our testing, the proportion of homologous contamination that could be removed from the simulated raw data decreased with increase in simulated contamination rates, and there was a significant negative correlation between them (*R^2^* = 0.391, *p* = .019). However, it remained possible to remove > 99% of homologous contaminations even when the simulated contamination rate reached 99% (Figure 6). It was notable that 99.9% homologous contamination was removed when the simulated contamination rate was only 95%. On average, 99.07% of contamination could be removed using our recommended screening method (Table S16), which was lower than that reported by many other ancient DNA studies (Sawyer et al., 2015; Schuenemann et al., 2017). No significant differences were found in endogenous DNA rates considering the different samples, different damage patterns, and different contamination rates: This demonstrated the universal property of our recommended method. Using the remaining endogenous ancient reads, we summarized a best combination with DeamNum = 1, DetectRange = 15, and DoubleOrSingle = or.

To test a potentially more effective filtering strategy, we further screened reads with G or A residues preceding the first base at 5’ end of the DNA fragments. This depurination screening decreased the homologous contamination rate to 2.25% (the initial contamination rates were from 20% to 99.9%), which meant that this method enables recovery of more endogenous DNA (Table S17). Similar to deamination screening, filtering effect showed no difference in relation to sample ages, which was largely due to weak correlation between depurination and samples ages. Sample age and the extent of DNA fragmentation were not significantly correlated. DNA fragments are usually heavily degraded due to depurination shortly after death (Dabney, Meyer et al., 2013; Sawyer et al., 2012). However, only 10%–40% of ancient DNA fragmentation is triggered by depurination although other factors can also result in DNA fragmentation. As such, it is difficult to identify more endogenous ancient reads by screening the DNA length. However, this has also been provided in our python script to support filtration by depurination and fragmentation as week filtering options (not recommended).

## 5 CONCLUSION

We found that BWA *mem* with the parameters ‐*k* = 19 and ‐*r* = 2.5 was comparable to BWA *aln* with MS parameters ( Schubert et al., 2012) when considering the recovery of ancient DNA, but had a significantly shorter running time than did BWA *aln* with MS parameters. For the recovery of endogenous DNA from ancient sequencing data with homologous contaminations, we recommend screening of reads with parameters: –DeamNum = 1, –DetectRange = 15, and –DoubleOrSingle = or, which could remove more than 99% of homologous DNA contaminations from the raw contaminated sequencing data. Overall, these recommendations for ancient DNA mapping and separation of endogenous DNA can benefit ancient DNA studies, especially for samples preserved under poor conditions.

## CONFLICT OF INTEREST

The authors declare no conflicts of interest.

## AUTHOR CONTRIBUTION

**Wenhao Xu:** Investigation (equal); Methodology (equal); Software (lead); Visualization (equal); Writing‐original draft (lead). **Yu Lin:** Validation (lead). **Keliang Zhao:** Conceptualization (equal). **Haimeng Li:** Validation (equal). **Yinpin Tian:** Data curation (equal). **Jacob Njaramba Ngatia:** Validation (equal); Writing‐original draft (equal); Writing‐review & editing (equal). **Yue Ma:** Validation (equal). **Sunil Kumar Sahu:** Writing‐review & editing (equal). **Huabing Guo:** Investigation (equal). **Xiaosen Guo:** Validation (equal). **Yanchun Xu:** Conceptualization (equal). **Huan Liu:** Conceptualization (equal). **Karsten Kristiansen:** Conceptualization (equal). **Tianming Lan:** Conceptualization (equal); Data curation (equal); Investigation (equal); Methodology (equal); Software (equal); Supervision (equal); Visualization (equal); Writing‐original draft (equal); Writing‐review & editing (equal). **Xinying Zhou:** Conceptualization (equal); Supervision (equal).

## Supporting information

Fig S1

Fig S2

Fig S3

Fig S4

Fig S5

Table S1

Table S2

Table S3

Table S4

Table S5

Table S6

Table S7

Table S8

Table S9

Table S10

Table S11

Table S12

Table S13

Table S14

Table S15

Table S16

Table S17

## Data Availability

Raw sequencing data of the ancient panda have been deposited to the CNSA (CNGB Nucleotide Sequence Archive) with accession number CNP0000732 (https://db.cngb.org/cnsa/).
