# Supplementary material for: An efficient pipeline for ancient DNA mapping and recovery of endogenous ancient DNA from whole‐genome sequencing data
Source: Ecol Evol. 2020 Dec 21;11(1):390–401. doi: 10.1002/ece3.7056 (PMC7790629; doi:10.1002/ece3.7056)
Supplement: Supplementary file 6 — Table S1 [file ECE3-11-390-s006.docx]

**Table S1. Information of reference genomes**

| **Species** | **No. of Reference genome** | **Version** | **Website** |
| --- | --- | --- | --- |
| *Bos primigenius* | bosTau8 | Jun. 2014 | http://hgdownload.cse.ucsc.edu/goldenPath/bosTau8/bigZips/bosTau8.fa.gz |
| *Capra aegagrus hircus* | GCF_001704415.1_ARS1 | Aug. 2016 | ftp://ftp.ncbi.nlm.nih.gov/genomes/all/GCF/001/704/415/GCF_001704415.1_ARS1/GCF_001704415.1_ARS1_genomic.fna.gz |
| *Homo sapiens* | GRCh38 | Dec. 2013 | http://hgdownload.cse.ucsc.edu/goldenPath/hg38/bigZips/hg38.fa.gz |
