# Supplementary material for: An efficient pipeline for ancient DNA mapping and recovery of endogenous ancient DNA from whole‐genome sequencing data
Source: Ecol Evol. 2020 Dec 21;11(1):390–401. doi: 10.1002/ece3.7056 (PMC7790629; doi:10.1002/ece3.7056)
Supplement: Supplementary file 7 — Table S2 [file ECE3-11-390-s007.docx]

**T****able S2. The parameter combinations we used in this study for filtering the homologous contaminations.**

| **At Least “DeamNum”**  **C-to-T or G-to-A Changes** | **Within the First or Last “DetectRange” Base Pair** | **Both Ends (And)/ Single End (Or)** |
| --- | --- | --- |
| 1 | 5 | And |
| 1 | 5 | Or |
| 1 | 10 | And |
| 1 | 10 | Or |
| 1 | 15 | And |
| 1 | 15 | Or |
| 2 | 5 | And |
| 2 | 5 | Or |
| 2 | 10 | And |
| 2 | 10 | Or |
| 2 | 15 | And |
| 2 | 15 | Or |
| 3 | 5 | And |
| 3 | 5 | Or |
| 3 | 10 | And |
| 3 | 10 | Or |
| 3 | 15 | And |
| 3 | 15 | Or |
