# Supplementary material for: An efficient pipeline for ancient DNA mapping and recovery of endogenous ancient DNA from whole‐genome sequencing data
Source: Ecol Evol. 2020 Dec 21;11(1):390–401. doi: 10.1002/ece3.7056 (PMC7790629; doi:10.1002/ece3.7056)
Supplement: Supplementary file 8 — Table S3 [file ECE3-11-390-s008.docx]

**Table S3. The information of 54 simulated data sets**

| Num | Contamination Reads Rate | Contamination Read Resource | Endogenous Reads Rate | Endogenous Reads Resource |
| --- | --- | --- | --- | --- |
| 1 | 20% | panda data | 80% | JK2911 |
| 2 | 20% | panda data | 80% | Villabruna |
| 3 | 20% | panda data | 80% | AfontovaCava 3 |
| 4 | 20% | panda data | 80% | Denisova_8 |
| 5 | 20% | panda data | 80% | British aurochs |
| 6 | 20% | panda data | 80% | Direkli5 |
| 7 | 40% | panda data | 60% | JK2911 |
| 8 | 40% | panda data | 60% | Villabruna |
| 9 | 40% | panda data | 60% | AfontovaCava 3 |
| 10 | 40% | panda data | 60% | Denisova_8 |
| 11 | 40% | panda data | 60% | British aurochs |
| 12 | 40% | panda data | 60% | Direkli5 |
| 13 | 60% | panda data | 40% | JK2911 |
| 14 | 60% | panda data | 40% | Villabruna |
| 15 | 60% | panda data | 40% | AfontovaCava 3 |
| 16 | 60% | panda data | 40% | Denisova_8 |
| 17 | 60% | panda data | 40% | British aurochs |
| 18 | 60% | panda data | 40% | Direkli5 |
| 19 | 80% | panda data | 20% | JK2911 |
| 20 | 80% | panda data | 20% | Villabruna |
| 21 | 80% | panda data | 20% | AfontovaCava 3 |
| 22 | 80% | panda data | 20% | Denisova_8 |
| 23 | 80% | panda data | 20% | British aurochs |
| 24 | 80% | panda data | 20% | Direkli5 |
| 25 | 90% | panda data | 10% | JK2911 |
| 26 | 90% | panda data | 10% | Villabruna |
| 27 | 90% | panda data | 10% | AfontovaCava 3 |
| 28 | 90% | panda data | 10% | Denisova_8 |
| 29 | 90% | panda data | 10% | British aurochs |
| 30 | 90% | panda data | 10% | Direkli5 |
| 31 | 95% | panda data | 5% | JK2911 |
| 32 | 95% | panda data | 5% | Villabruna |
| 33 | 95% | panda data | 5% | AfontovaCava 3 |
| 34 | 95% | panda data | 5% | Denisova_8 |
| 35 | 95% | panda data | 5% | British aurochs |
| 36 | 95% | panda data | 5% | Direkli5 |
| 37 | 99% | panda data | 1% | JK2911 |
| 38 | 99% | panda data | 1% | Villabruna |
| 39 | 99% | panda data | 1% | AfontovaCava 3 |
| 40 | 99% | panda data | 1% | Denisova_8 |
| 41 | 99% | panda data | 1% | British aurochs |
| 42 | 99% | panda data | 1% | Direkli5 |
| 43 | 99.5% | panda data | 0.5% | JK2911 |
| 44 | 99.5% | panda data | 0.5% | Villabruna |
| 45 | 99.5% | panda data | 0.5% | AfontovaCava 3 |
| 46 | 99.5% | panda data | 0.5% | Denisova_8 |
| 47 | 99.5% | panda data | 0.5% | British aurochs |
| 48 | 99.5% | panda data | 0.5% | Direkli5 |
| 49 | 99.9% | panda data | 0.1% | JK2911 |
| 50 | 99.9% | panda data | 0.1% | Villabruna |
| 51 | 99.9% | panda data | 0.1% | AfontovaCava 3 |
| 52 | 99.9% | panda data | 0.1% | Denisova_8 |
| 53 | 99.9% | panda data | 0.1% | British aurochs |
| 54 | 99.9% | panda data | 0.1% | Direkli5 |
| 55 | 20% | Hg38 | 80% | JK2911 |
| 56 | 20% | Hg38 | 80% | Villabruna |
| 57 | 20% | Hg38 | 80% | AfontovaCava 3 |
| 58 | 20% | Hg38 | 80% | Denisova_8 |
| 59 | 40% | Hg38 | 60% | JK2911 |
| 60 | 40% | Hg38 | 60% | Villabruna |
| 61 | 40% | Hg38 | 60% | AfontovaCava 3 |
| 62 | 40% | Hg38 | 60% | Denisova_8 |
| 63 | 60% | Hg38 | 40% | JK2911 |
| 64 | 60% | Hg38 | 40% | Villabruna |
| 65 | 60% | Hg38 | 40% | AfontovaCava 3 |
| 66 | 60% | Hg38 | 40% | Denisova_8 |
| 67 | 80% | Hg38 | 20% | JK2911 |
| 68 | 80% | Hg38 | 20% | Villabruna |
| 69 | 80% | Hg38 | 20% | AfontovaCava 3 |
| 70 | 80% | Hg38 | 20% | Denisova_8 |
| 71 | 90% | Hg38 | 10% | JK2911 |
| 72 | 90% | Hg38 | 10% | Villabruna |
| 73 | 90% | Hg38 | 10% | AfontovaCava 3 |
| 74 | 90% | Hg38 | 10% | Denisova_8 |
| 75 | 95% | Hg38 | 5% | JK2911 |
| 76 | 95% | Hg38 | 5% | Villabruna |
| 77 | 95% | Hg38 | 5% | AfontovaCava 3 |
| 78 | 95% | Hg38 | 5% | Denisova_8 |
| 79 | 99% | Hg38 | 1% | JK2911 |
| 80 | 99% | Hg38 | 1% | Villabruna |
| 81 | 99% | Hg38 | 1% | AfontovaCava 3 |
| 82 | 99% | Hg38 | 1% | Denisova_8 |
| 83 | 99.5% | Hg38 | 0.5% | JK2911 |
| 84 | 99.5% | Hg38 | 0.5% | Villabruna |
| 85 | 99.5% | Hg38 | 0.5% | AfontovaCava 3 |
| 86 | 99.5% | Hg38 | 0.5% | Denisova_8 |
| 87 | 99.9% | Hg38 | 0.1% | JK2911 |
| 88 | 99.9% | Hg38 | 0.1% | Villabruna |
| 89 | 99.9% | Hg38 | 0.1% | AfontovaCava 3 |
| 90 | 99.9% | Hg38 | 0.1% | Denisova_8 |

#panda data: we sequenced the historic sample (~100 years old) by DIPSEQ-T1 platform. And, raw reads were n mapped by blast using the nucleotide database to obtain the true proportion of contaminated reads
