# Supplementary material for: An efficient pipeline for ancient DNA mapping and recovery of endogenous ancient DNA from whole‐genome sequencing data
Source: Ecol Evol. 2020 Dec 21;11(1):390–401. doi: 10.1002/ece3.7056 (PMC7790629; doi:10.1002/ece3.7056)
Supplement: Supplementary file 9 — Table S4 [file ECE3-11-390-s009.docx]

**Table S4. The mean and median values of CRT, LRE and MT of MS and BWA *mem* with different simulated contamination rates**

| **Simulated Contamination Rates** | | **20%** | **40%** | **60%** | **80%** | **90%** | **95%** | **99%** | **99.5%** | **99.9%** |
| --- | --- | --- | --- | --- | --- | --- | --- | --- | --- | --- |
| **CRT (%)** | | | | | | | | | | |
| MS | Mean | 0.01 | 0.03 | 0.08 | 0.20 | 0.50 | 0.95 | 15.62 | 8.84 | 33.12 |
|  | Median | 0.01 | 0.03 | 0.08 | 0.19 | 0.43 | 0.97 | 9.69 | 8.69 | 33.31 |
| BWA *mem* | Mean | 0.02 | 0.04 | 0.09 | 0.23 | 0.54 | 1.14 | 17.93 | 10.81 | 37.28 |
|  | Median | 0.01 | 0.04 | 0.09 | 0.23 | 0.56 | 1.12 | 10.63 | 10.63 | 37.40 |
| **LRE (%)** | | | | | | | | | | |
| MS | Mean | 21.72 | 21.69 | 21.70 | 21.68 | 21.67 | 21.71 | 21.50 | 21.78 | 21.18 |
|  | Median | 21.03 | 20.94 | 21.02 | 21.00 | 20.91 | 20.98 | 22.01 | 21.49 | 22.65 |
| BWA *mem* | Mean | 22.40 | 22.36 | 22.38 | 22.38 | 22.35 | 22.40 | 22.10 | 22.51 | 21.48 |
|  | Median | 23.34 | 23.20 | 23.31 | 23.28 | 23.11 | 23.35 | 22.69 | 22.69 | 22.90 |
| **MT (mins)** | | | | | | | | | | |
| MS | Mean | 14.33 | 11.22 | 20.70 | 22.52 | 19.33 | 21.62 | 24.48 | 29.08 | 20.43 |
|  | Median | 11.60 | 11.25 | 19.45 | 27.20 | 17.3 | 21.40 | 21.55 | 23.7 | 20.85 |
| BWA *mem* | Mean | 0.01 | 0.03 | 0.08 | 0.20 | 0.50 | 0.95 | 15.62 | 8.84 | 33.12 |
|  | Median | 0.01 | 0.03 | 0.08 | 0.19 | 0.43 | 0.97 | 9.69 | 8.69 | 33.31 |

# MS means BWA *aln -l 1024 -n 0.03*; BWA *mem* means BWA *mem* with default parameters.
