# Supplementary material for: An efficient pipeline for ancient DNA mapping and recovery of endogenous ancient DNA from whole‐genome sequencing data
Source: Ecol Evol. 2020 Dec 21;11(1):390–401. doi: 10.1002/ece3.7056 (PMC7790629; doi:10.1002/ece3.7056)
Supplement: Supplementary file 10 — Table S5 [file ECE3-11-390-s010.docx]

**Table S5.** **Differences among** **CRT, LRE and MT in mapping results from BWA *aln* with MS and BWA *mem* with default parameters** **evaluated by Repeated Measures ANOVA**

|  | Groups | *df* | *F* Value | Adj *P* Value |
| --- | --- | --- | --- | --- |
| CRT | Mapping methods | 1 | 1.42 | 0.2870 |
| LRE | Mapping methods | 1 | 0.44 | 0.5344 |
| MT | Mapping methods | 1 | 41.57 | 0.0013 |

**#Mapping methods**: the two mapping algorithms we used in this study (BWA *aln* with MS parameters and BWA *mem* with default parameters).

***df***: degrees of freedom.

**Adj *P* Value**: adjusted *P* value by Greenhouse-Geisser (G-G) method.
