# Supplementary material for: An efficient pipeline for ancient DNA mapping and recovery of endogenous ancient DNA from whole‐genome sequencing data
Source: Ecol Evol. 2020 Dec 21;11(1):390–401. doi: 10.1002/ece3.7056 (PMC7790629; doi:10.1002/ece3.7056)
Supplement: Supplementary file 11 — Table S6 [file ECE3-11-390-s011.docx]

**Table S6. the average CRT/LRE/MT of 6 samples by using BWA *mem* and MS**

| **Samples** | **Mapping algorithm** | | **CRT (%)** | **LRE (%)** | **MT(mins)** |
| --- | --- | --- | --- | --- | --- |
| AfontovaCave3 | BWA *mem* | Mean | 7.08144015244 | 24.9344234189 | 2.722 |
|  |  | N | 9 | 9 | 9 |
|  |  | Std. Deviation | 13.793154496873 | .45079535727 | 1.6687 |
|  | MS | Mean | 4.66842839778 | 26.0998691456 | 24.633 |
|  |  | N | 9 | 9 | 9 |
|  |  | Std. Deviation | 9.540569279335 | .33374640667 | 7.7701 |
|  | Total | Mean | 5.87493427511 | 25.5171462822 | 13.678 |
|  |  | N | 18 | 18 | 18 |
|  |  | Std. Deviation | 11.571746527804 | .71245275521 | 12.5222 |
| British aurochs | BWA *mem* | Mean | 5.76643469089 | 19.2790580989 | 1.833 |
|  |  | N | 9 | 9 | 9 |
|  |  | Std. Deviation | 11.448095890542 | .61370655958 | 1.1489 |
|  | MS | Mean | 6.59616000289 | 17.3446601878 | 24.722 |
|  |  | N | 9 | 9 | 9 |
|  |  | Std. Deviation | 12.780092809528 | .53057101620 | 14.2984 |
|  | Total | Mean | 6.18129734689 | 18.3118591433 | 13.278 |
|  |  | N | 18 | 18 | 18 |
|  |  | Std. Deviation | 11.777883991352 | 1.14026982125 | 15.3463 |
| Denisova8 | BWA *mem* | Mean | 5.25951179856 | 23.6906359867 | 2.633 |
|  |  | N | 9 | 9 | 9 |
|  |  | Std. Deviation | 10.511659570423 | .70979359467 | 1.4534 |
|  | MS | Mean | 6.19056185311 | 27.1056420111 | 29.089 |
|  |  | N | 9 | 9 | 9 |
|  |  | Std. Deviation | 12.004086828161 | .75072467544 | 15.3222 |
|  | Total | Mean | 5.72503682583 | 25.3981389989 | 15.861 |
|  |  | N | 18 | 18 | 18 |
|  |  | Std. Deviation | 10.956184243287 | 1.89456426069 | 17.2261 |
| Direkli5 | BWA *mem* | Mean | 5.76317203978 | 24.2629295567 | 2.689 |
|  |  | N | 9 | 9 | 9 |
|  |  | Std. Deviation | 11.525937975893 | .41521563716 | 1.4786 |
|  | MS | Mean | 6.02296819733 | 22.4633875667 | 14.433 |
|  |  | N | 9 | 9 | 9 |
|  |  | Std. Deviation | 12.173701058608 | .43327252673 | 9.5984 |
|  | Total | Mean | 5.89307011856 | 23.3631585617 | 8.561 |
|  |  | N | 18 | 18 | 18 |
|  |  | Std. Deviation | 11.501083510858 | 1.01325375848 | 8.9942 |
| JK2911 | BWA *mem* | Mean | 6.77986114822 | 18.9843047344 | 2.311 |
|  |  | N | 9 | 9 | 9 |
|  |  | Std. Deviation | 13.076494744141 | .90678712101 | 1.3815 |
|  | MS | Mean | 4.13716273822 | 16.8511653889 | 12.200 |
|  |  | N | 9 | 9 | 9 |
|  |  | Std. Deviation | 8.690763982251 | .76767213907 | 6.1292 |
|  | Total | Mean | 5.45851194322 | 17.9177350617 | 7.256 |
|  |  | N | 18 | 18 | 18 |
|  |  | Std. Deviation | 10.856334811306 | 1.36702645896 | 6.6680 |
| Villabruna | BWA *mem* | Mean | 6.59566872000 | 22.5549356700 | 2.822 |
|  |  | N | 9 | 9 | 9 |
|  |  | Std. Deviation | 12.959357802639 | .39444418739 | .9972 |
|  | MS | Mean | 4.78320962678 | 19.9097876856 | 17.022 |
|  |  | N | 9 | 9 | 9 |
|  |  | Std. Deviation | 9.808200325473 | .79275118119 | 6.1161 |
|  | Total | Mean | 5.68943917339 | 21.2323616778 | 9.922 |
|  |  | N | 18 | 18 | 18 |
|  |  | Std. Deviation | 11.188093782053 | 1.49032094029 | 8.4526 |

# MS means BWA *aln -l 1024 -n 0.03*; BWA *mem* means BWA *mem* with default parameters.
