# Supplementary material for: An efficient pipeline for ancient DNA mapping and recovery of endogenous ancient DNA from whole‐genome sequencing data
Source: Ecol Evol. 2020 Dec 21;11(1):390–401. doi: 10.1002/ece3.7056 (PMC7790629; doi:10.1002/ece3.7056)
Supplement: Supplementary file 12 — Table S7 [file ECE3-11-390-s012.docx]

**Table S7.** **Differences among CRT, LRE and MT in mapping results from BWA *mem* with different “-k” values evaluated by Repeated Measures ANOVA.**

|  | Groups | *df* | *F* Value | Adj *P* Value |
| --- | --- | --- | --- | --- |
| CRT | Mapping methods | 4 | 644.61 | <0.0001 |
| LRE | Mapping methods | 4 | 17.99 | 0.0057 |
| MT | Mapping methods | 4 | 146.75 | <0.0001 |

**#Mapping methods**: BWA *mem* with different “-k” values we used in this study. (“-k=9”, “-k=14”, “-k=19”, “-k=24”, “-k=29”,)

***df***: degrees of freedom.

**Adj *P* Value**: adjusted *P* value by Greenhouse-Geisser (G-G) method.
