# Supplementary material for: An efficient pipeline for ancient DNA mapping and recovery of endogenous ancient DNA from whole‐genome sequencing data
Source: Ecol Evol. 2020 Dec 21;11(1):390–401. doi: 10.1002/ece3.7056 (PMC7790629; doi:10.1002/ece3.7056)
Supplement: Supplementary file 13 — Table S8 [file ECE3-11-390-s013.docx]

**Table S8. LSD analysis for CRT/LRE/MT of mapping results from BWA *mem* with different “-k” values.**

| **Dependent Variable** | | | | | **Mean Difference (I-J)** | **Std. Error** | **Sig.** | **95% Confidence Interval** | |
| --- | --- | --- | --- | --- | --- | --- | --- | --- | --- |
|  | **I** | | **J** | |  |  |  | **Lower Bound** | **Upper Bound** |
| CRT (%) | | -k 9 | | -k 14 | 0.07136 | 2.14168 | 0.97344 | -4.14552 | 4.28825 |
|  |  |  |  | -k 19 | 0.20041 | 2.14168 | 0.92552 | -4.01648 | 4.41729 |
|  |  |  |  | -k 24 | 0.47441 | 2.14168 | 0.82486 | -3.74247 | 4.69129 |
|  |  |  |  | -k 29 | 2.22180 | 2.14168 | 0.30049 | -1.99508 | 6.43868 |
|  |  | -k 14 | | -k 9 | -0.07136 | 2.14168 | 0.97344 | -4.28825 | 4.14552 |
|  |  |  |  | -k 19 | 0.12904 | 2.14168 | 0.95200 | -4.08784 | 4.34592 |
|  |  |  |  | -k 24 | 0.40305 | 2.14168 | 0.85087 | -3.81383 | 4.61993 |
|  |  |  |  | -k 29 | 2.15043 | 2.14168 | 0.31625 | -2.06645 | 6.36731 |
|  |  | -k 19 | | -k 9 | -0.20041 | 2.14168 | 0.92552 | -4.41729 | 4.01648 |
|  |  |  |  | -k 14 | -0.12904 | 2.14168 | 0.95200 | -4.34592 | 4.08784 |
|  |  |  |  | -k 24 | 0.27401 | 2.14168 | 0.89829 | -3.94288 | 4.49089 |
|  |  |  |  | -k 29 | 2.02139 | 2.14168 | 0.34611 | -2.19549 | 6.23827 |
|  |  | -k 24 | | -k 9 | -0.47441 | 2.14168 | 0.82486 | -4.69129 | 3.74247 |
|  |  |  |  | -k 14 | -0.40305 | 2.14168 | 0.85087 | -4.61993 | 3.81383 |
|  |  |  |  | -k 19 | -0.27401 | 2.14168 | 0.89829 | -4.49089 | 3.94288 |
|  |  |  |  | -k 29 | 1.74739 | 2.14168 | 0.41529 | -2.46950 | 5.96427 |
|  |  | -k 29 | | -k 9 | -2.22180 | 2.14168 | 0.30049 | -6.43868 | 1.99508 |
|  |  |  |  | -k 14 | -2.15043 | 2.14168 | 0.31625 | -6.36731 | 2.06645 |
|  |  |  |  | -k 19 | -2.02139 | 2.14168 | 0.34611 | -6.23827 | 2.19549 |
|  |  |  |  | -k 24 | -1.74739 | 2.14168 | 0.41529 | -5.96427 | 2.46950 |
| LRE (%) | | -k 9 | | -k 14 | -0.05103 | 0.56843 | 0.92854 | -1.17023 | 1.06818 |
|  |  |  |  | -k 19 | -0.19537 | 0.56843 | 0.73134 | -1.31458 | 0.92384 |
|  |  |  |  | -k 24 | -0.83545 | 0.56843 | 0.14281 | -1.95465 | 0.28376 |
|  |  |  |  | -k 29 | -4.85069* | 0.56843 | 0.00000 | -5.96990 | -3.73149 |
|  |  | -k 14 | | -k 9 | 0.05103 | 0.56843 | 0.92854 | -1.06818 | 1.17023 |
|  |  |  |  | -k 19 | -0.14434 | 0.56843 | 0.79975 | -1.26355 | 0.97487 |
|  |  |  |  | -k 24 | -0.78442 | 0.56843 | 0.16876 | -1.90362 | 0.33479 |
|  |  |  |  | -k 29 | -4.79966* | 0.56843 | 0.00000 | -5.91887 | -3.68046 |
|  |  | -k 19 | | -k 9 | 0.19537 | 0.56843 | 0.73134 | -0.92384 | 1.31458 |
|  |  |  |  | -k 14 | 0.14434 | 0.56843 | 0.79975 | -0.97487 | 1.26355 |
|  |  |  |  | -k 24 | -0.64008 | 0.56843 | 0.26116 | -1.75928 | 0.47913 |
|  |  |  |  | -k 29 | -4.65532* | 0.56843 | 0.00000 | -5.77453 | -3.53612 |
|  |  | -k 24 | | -k 9 | 0.83545 | 0.56843 | 0.14281 | -0.28376 | 1.95465 |
|  |  |  |  | -k 14 | 0.78442 | 0.56843 | 0.16876 | -0.33479 | 1.90362 |
|  |  |  |  | -k 19 | 0.64008 | 0.56843 | 0.26116 | -0.47913 | 1.75928 |
|  |  |  |  | -k 29 | -4.01524* | 0.56843 | 0.00000 | -5.13446 | -2.89604 |
|  |  | -k 29 | | -k 9 | 4.85069* | 0.56843 | 0.00000 | 3.73149 | 5.96990 |
|  |  |  |  | -k 14 | 4.79966* | 0.56843 | 0.00000 | 3.68046 | 5.91887 |
|  |  |  |  | -k 19 | 4.65532* | 0.56843 | 0.00000 | 3.53612 | 5.77453 |
|  |  |  |  | -k 24 | 4.01524* | 0.56843 | 0.00000 | 2.89604 | 5.13446 |
| MT (mins) | | -k 9 | | -k 14 | 50.2778* | 2.1811 | 0.000 | 45.983 | 54.572 |
|  |  |  |  | -k 19 | 55.9019* | 2.1811 | 0.000 | 51.607 | 60.196 |
|  |  |  |  | -k 24 | 56.4444* | 2.1811 | 0.000 | 52.150 | 60.739 |
|  |  |  |  | -k 29 | 56.4833* | 2.1811 | 0.000 | 52.189 | 60.778 |
|  |  | -k 14 | | -k 9 | -50.2778* | 2.1811 | 0.000 | -54.572 | -45.983 |
|  |  |  |  | -k 19 | 5.6241* | 2.1811 | 0.010 | 1.330 | 9.919 |
|  |  |  |  | -k 24 | 6.1667* | 2.1811 | 0.005 | 1.872 | 10.461 |
|  |  |  |  | -k 29 | 6.2056* | 2.1811 | 0.005 | 1.911 | 10.500 |
|  |  | -k 19 | | -k 9 | -55.9019* | 2.1811 | 0.000 | -60.196 | -51.607 |
|  |  |  |  | -k 14 | -5.6241* | 2.1811 | 0.010 | -9.919 | -1.330 |
|  |  |  |  | -k 24 | 0.5426 | 2.1811 | 0.804 | -3.752 | 4.837 |
|  |  |  |  | -k 29 | 0.5815 | 2.1811 | 0.790 | -3.713 | 4.876 |
|  |  | -k 24 | | -k 9 | -56.4444* | 2.1811 | 0.000 | -60.739 | -52.150 |
|  |  |  |  | -k 14 | -6.1667* | 2.1811 | 0.005 | -10.461 | -1.872 |
|  |  |  |  | -k 19 | -0.5426 | 2.1811 | 0.804 | -4.837 | 3.752 |
|  |  |  |  | -k 29 | 0.0389 | 2.1811 | 0.986 | -4.256 | 4.333 |
|  |  | -k 29 | | -k 9 | -56.4833* | 2.1811 | 0.000 | -60.778 | -52.189 |
|  |  |  |  | -k 14 | -6.2056* | 2.1811 | 0.005 | -10.500 | -1.911 |
|  |  |  |  | -k 19 | -0.5815 | 2.1811 | 0.790 | -4.876 | 3.713 |
|  |  |  |  | -k 24 | -0.0389 | 2.1811 | 0.986 | -4.333 | 4.256 |
| #The mean difference is significant at the 0.05 level.  **LSD** uses t-tests to perform all pairwise comparisons between group means. No adjustment is made to the error rate for multiple comparisons. Used for pairwise comparison. This test does not control the overall probability of rejecting the hypotheses that some pairs of means are different, while in fact they are equal. | | | | | | | | | |

**Mean Difference (I-J)** = (the mean of dependent variable_(I)_) – (the mean of dependent variable_(J)_)
