# Supplementary material for: An efficient pipeline for ancient DNA mapping and recovery of endogenous ancient DNA from whole‐genome sequencing data
Source: Ecol Evol. 2020 Dec 21;11(1):390–401. doi: 10.1002/ece3.7056 (PMC7790629; doi:10.1002/ece3.7056)
Supplement: Supplementary file 14 — Table S9 [file ECE3-11-390-s014.docx]

**Table S9.** **Differences among CRT, LRE and MT in mapping results from BWA *mem* with different “-r” values evaluated by Repeated Measures ANOVA**

|  | Groups | *df* | *F* Value | Adj *P* Value |
| --- | --- | --- | --- | --- |
| CRT | Mapping methods | 4 | 392.45 | <0.0001 |
| LRE | Mapping methods | 4 | 392.45 | 0.0010 |
| MT | Mapping methods | 4 | 9.19 | 0.0002 |

**#Mapping methods**: BWA *mem* with different “-r” values we used in this study (“-r 0.5”, “-r 1.0”, “-r 1.5”, “-r 2.0”, “-r 2.5”).

***df***: degrees of freedom.

**Adj *P* Value**: adjusted *P* value by Greenhouse-Geisser (G-G) method.
