# Supplementary material for: An efficient pipeline for ancient DNA mapping and recovery of endogenous ancient DNA from whole‐genome sequencing data
Source: Ecol Evol. 2020 Dec 21;11(1):390–401. doi: 10.1002/ece3.7056 (PMC7790629; doi:10.1002/ece3.7056)
Supplement: Supplementary file 16 — Table S11 [file ECE3-11-390-s016.docx]

**Table S11. The average of CRT and LRE calculated by using the mapping results with different filtering options**

**A.**

| **Option: DetectRange** | **CRT(%)** | **LRE(%)** |
| --- | --- | --- |
| 5 | 2.03 | 96.95 |
| 10 | 2.25 | 95.96 |
| 15 | 1.57 | 95.31 |
| Total | 1.90 | 96.08 |

**B.**

| **Option: DeamNum** | **CRT(%)** | **LRE(%)** |
| --- | --- | --- |
| 1 | 2.85 | 88.87 |
| 2 | 0.29 | 99.37 |
| 3 | 0.00 | 99.98 |
| Total | 1.90 | 96.08 |

**C.**

| **Option: DoubleOrSingle** | **CRT(%)** | **LRE(%)** |
| --- | --- | --- |
| and | 5.14 | 99.95 |
| or | 0.50 | 92.20 |
| Total | 1.90 | 96.08 |

# “-DeamNum” means screening reads with at least x C-to-T or G-to-A mutations at ends of DNA fragments.

“-DetectRange” means screening reads with C-to-T or G-to-A mutations within the first or last x base pair.

“-DoubleOrSingle” means screening reads with C-to-T or G-to-A mutations at 3’ and/or 5’ ends.
