# Supplementary material for: An efficient pipeline for ancient DNA mapping and recovery of endogenous ancient DNA from whole‐genome sequencing data
Source: Ecol Evol. 2020 Dec 21;11(1):390–401. doi: 10.1002/ece3.7056 (PMC7790629; doi:10.1002/ece3.7056)
Supplement: Supplementary file 17 — Table S12 [file ECE3-11-390-s017.docx]

**Table S12. Differences among CRT and LRE evaluated by Repeated Measures ANOVA** **when applied the different “DetectRange” for filtering the homologous contaminations**

means screening reads with C-to-T or G-to-A mutations within the first or last Y base pair.

|  | Groups | *df* | *F* Value | Adj *P* Value |
| --- | --- | --- | --- | --- |
| CRT | DetectRange | 2 | 3.27 | 0.1097 |
| LRE | DetectRange | 2 | 1.11 | 0.3893 |

**# DetectRange** means screening reads with C-to-T or G-to-A mutations within the first or last x base pair (“- DetectRange=5”, “- DetectRange=10”, “- DetectRange=15”).

***df***: degrees of freedom.

**Adj *P* Value**: adjusted *P* value by Greenhouse-Geisser (G-G) method.
