# Supplementary material for: An efficient pipeline for ancient DNA mapping and recovery of endogenous ancient DNA from whole‐genome sequencing data
Source: Ecol Evol. 2020 Dec 21;11(1):390–401. doi: 10.1002/ece3.7056 (PMC7790629; doi:10.1002/ece3.7056)
Supplement: Supplementary file 18 — Table S13 [file ECE3-11-390-s018.docx]

**Table S13. Differences among CRT and LRE evaluated by Repeated Measures ANOVA when applied the different “DeamNum” for filtering the homologous contaminations**

|  | Groups | *df* | *F* Value | Adj *P* Value |
| --- | --- | --- | --- | --- |
| CRT | DeamNum | 2 | 26.01 | 0.0011 |
| LRE | DeamNum | 2 | 24.03 | 0.0152 |

**# DeamNum** means screening reads with at least x C-to-T or G-to-A mutations at ends of DNA fragments (“-DeamNum =1”, “-DeamNum =2”, “-DeamNum =3”).

***df***: degrees of freedom.

**Adj *P* Value**: adjusted *P* value by Greenhouse-Geisser (G-G) method.
