# Supplementary material for: An efficient pipeline for ancient DNA mapping and recovery of endogenous ancient DNA from whole‐genome sequencing data
Source: Ecol Evol. 2020 Dec 21;11(1):390–401. doi: 10.1002/ece3.7056 (PMC7790629; doi:10.1002/ece3.7056)
Supplement: Supplementary file 19 — Table S14 [file ECE3-11-390-s019.docx]

**Table S14. Differences among CRT and LRE evaluated by Repeated Measures ANOVA when applied the different “DoubleOrSingle” for filtering the homologous contaminations**

|  | Groups | *df* | *F* Value | Adj *P* Value |
| --- | --- | --- | --- | --- |
| CRT | DoubleOrSingle | 1 | 44.97 | 0.0068 |
| LRE | DoubleOrSingle | 1 | 7.20 | 0.0748 |

**# DoubleOrSingle** means screening reads with C-to-T or G-to-A mutations at 3’ and/or 5’ ends (“-DoubleOrSingle=or”, “-DoubleOrSingle=and”).

***df***: degrees of freedom.

**Adj *P* Value**: adjusted *P* value by Greenhouse-Geisser (G-G) method.
