# Supplementary material for: An efficient pipeline for ancient DNA mapping and recovery of endogenous ancient DNA from whole‐genome sequencing data
Source: Ecol Evol. 2020 Dec 21;11(1):390–401. doi: 10.1002/ece3.7056 (PMC7790629; doi:10.1002/ece3.7056)
Supplement: Supplementary file 20 — Table S15 [file ECE3-11-390-s020.docx]

**Table S15. The mean values of CRT and LRE in results calculated by PMDtools with different “-threshold” values**

| **“-threshold”** | **CRT(%)** | **LRE(%)** |
| --- | --- | --- |
| 1 | 0.77 | 52.44 |
| 2 | 0.56 | 68.03 |
| 3 | 0.49 | 73.82 |
| 4 | 0.46 | 80.19 |
| 5 | 0.43 | 89.01 |
