# Supplementary material for: An efficient pipeline for ancient DNA mapping and recovery of endogenous ancient DNA from whole‐genome sequencing data
Source: Ecol Evol. 2020 Dec 21;11(1):390–401. doi: 10.1002/ece3.7056 (PMC7790629; doi:10.1002/ece3.7056)
Supplement: Supplementary file 21 — Table S16 [file ECE3-11-390-s021.docx]

**Table S16. The mean values of homologous contamination rate after filtering by use parameter combinations of “–DeamNum=15 –DetectRange=1 –DoubleOrSingle=or”**

| **Simulated Contamination Rate (%)** | **Homologous Contamination Rate (%)** |
| --- | --- |
| 20 | 0.002 |
| 40 | 0.004 |
| 60 | 0.012 |
| 80 | 0.017 |
| 90 | 0.059 |
| 95 | 0.137 |
| 99 | 0.782 |
| 99.5 | 1.102 |
| 99.9 | 6.242 |
| Total | 0.929 |
