# Supplementary material for: An efficient pipeline for ancient DNA mapping and recovery of endogenous ancient DNA from whole‐genome sequencing data
Source: Ecol Evol. 2020 Dec 21;11(1):390–401. doi: 10.1002/ece3.7056 (PMC7790629; doi:10.1002/ece3.7056)
Supplement: Supplementary file 22 — Table S17 [file ECE3-11-390-s022.docx]

**Table S17. The mean values of CRT after filtering according to the depurination information.**

| **Simulated Contamination Rate (%)** | **CRT (%)** |
| --- | --- |
| 20 | 0.005 |
| 40 | 0.010 |
| 60 | 0.025 |
| 80 | 0.069 |
| 90 | 0.166 |
| 95 | 0.303 |
| 99 | 1.728 |
| 99.5 | 3.200 |
| 99.9 | 14.723 |
| Total | 2.248 |
